# Management of Intra-Abdominal Candidiasis in Intensive Care Setting: A Narrative Review

**DOI:** 10.3390/jof11050362

**Published:** 2025-05-06

**Authors:** Marco Marotta Pais, Rafael Zaragoza, Ignacio Martín-Loeches, Frederic F. Gómez-Bertomeu, Alejandro Rodríguez

**Affiliations:** 1 Critical Care Department, Hospital Universitari de Tarragona Joan XXIII, Mallafré Guasch 4, 43007 Tarragona, Spain; mmarottapais@gmail.com; 2 Department of Medicine and Surgery, Faculty of Medicine and Health Sciences, Rovira & Virgili University, 43005 Tarragona, Spain; ffgomez.hj23.ics@gencat.cat; 3 Critical Care Department, Hospital Universitario Dr. Peset, Av. Gaspar Aguilar 90, 46017 Valencia, Spain; zaragoza_raf@gva.es; 4 Department of Intensive Care Medicine, Multidisciplinary Intensive Care Research Organization (MICRO), St James’ Hospital, D08 NHY1 Dublin, Ireland; drmartinloeches@gmail.com; 5 Microbiology/Clinical Analysis Laboratory, Hospital Universitari de Tarragona Joan XXIII, Mallafré Guasch 4, 43007 Tarragona, Spain; 6 IISPV (Instituto de Investigación Sanitaria Pere Virgili), 43005 Tarragona, Spain; 7 Centre for Biomedical Research in Infectious Diseases Network (CIBERINFEC), 28220 Madrid, Spain; 8 Centre for Biomedical Research Network Respiratory Diseases (CIBERES), 43005 Tarragona, Spain; 9 Department of Basic Medical Sciences, Faculty of Medicine and Health Sciences, Rovira & Virgili University, 43201 Reus, Spain

**Keywords:** invasive candidiasis, antifungal treatment, diagnosis, outcome

## Abstract

Intra-abdominal candidiasis (IAC), with or without candidemia, is a common condition in patients in intensive care units (ICUs). Early diagnosis of IAC remains a challenge for clinicians despite new biomarkers. Early and appropriate antifungal treatment, which is associated with better clinical outcomes, is negatively affected by the increased isolation of non-albicans *Candida* strains that are resistant to the commonly used azoles and echinocandins. Based on the pharmacokinetic (PK) and pharmacodynamic (PD) properties of the different treatment options, liposomal amphotericin B, rezafungin or high doses of anidulafungin appear to be the most appropriate first-line options for complicated IAC in ICUs.

## 1. Introduction

Intra-abdominal candidiasis (IAC), with or without associated candidemia, is caused by overgrowth of *Candida* spp. in the abdominal cavity (see Table 1), and its incidence has increased in recent decades. IAC mainly affects critically ill patients undergoing major abdominal surgery, especially those with complex surgical cases or immunocompromised individuals [1,2,3,4,5].

Despite advances in prevention strategies, diagnostic tools and therapeutic approaches, IAC remains associated with high mortality rates, reaching up to 60% [6,7] This highlights the critical need for ongoing evaluation and optimization of management strategies [1,2,3,4,5].

The aim of this review is to update key concepts related to the changing epidemiology of IAC towards non-albicans *Candida* strains and the impact this situation may have on appropriate antifungal treatment, as well as to update concepts on the impact of *Candida* spp. in the peritoneal cavity, the risk factors associated with its presence and the interactions with bacteria leading to increased mortality. Finally, basic concepts of different antifungal treatment strategies based on pharmacokinetic/pharmacodynamic (PK/PD) parameters and antifungal susceptibility will be developed in order to develop an empirical treatment algorithm adapted to clinical realities.

**Table 1:** Classification of intra-abdominal candidiasis (IAC) types (modified from reference [8]).

Primary Peritonitis	Peritoneal inflammation associated with the isolation of *Candida* spp. in the absence of gastrointestinal perforation or any other visceral process that justifies it.
Secondary Peritonitis	Peritoneal infection by *Candida* spp. because of a pathological process or gastrointestinal perforation.
Intra-Abdominal Abscess of Gastrointestinal Origin	Isolation of *Candida* spp. in a collection of purulent material arising from secondary peritonitis.
Secondary Peritonitis of Hepatobiliary or Pancreatic Origin	Peritoneal infection by *Candida* spp. resulting from a pathological process of the liver, gallbladder, bile ducts or pancreas.
Intra-Abdominal Abscess of Hepatobiliary or Pancreatic Origin	Abscess with isolation of *Candida* spp. resulting from a pathological process of the liver, gallbladder, bile ducts or pancreas. Infected bilomas, pancreatic pseudocysts or other (peri)pancreatic collections are also classified as abscesses.
Infected Pancreatic Necrosis	Isolation of *Candida* spp. in non-viable pancreatic tissue as a consequence of pancreatitis.
Cholecystitis, Cholangitis	Isolation of *Candida* spp. in material from the gallbladder or biliary tract.
Recurrent IAC	Isolation of *Candida* spp. appearing after the apparent resolution of clinical or radiological findings of an initial infection.
Persistent IAC	Isolation of *Candida* spp. in a new intra-abdominal sample 48 h after adequate source control and appropriate antifungal treatment.

## 2. Epidemiology

There are at least 15 different *Candida* species that cause human disease, but over 95% of invasive disease is caused by the 6 most common pathogens: *Candida albicans*, *Candida glabrata*, *Candida tropicalis*, *Candida parapsilosis*, *Candida krusei* and, in some regions, *Candida auris.* Severe infections due to these organisms are collectively referred to as invasive candidiasis (IC) [8]. In recent decades, there has been a notable epidemiological shift with an increase in non-albicans *Candida* species. This shift from *C. albicans* to non-albicans species has significant clinical implications, particularly in terms of treatment efficacy, patient survival and antifungal resistance. Non-albicans species such as *C. glabrata*, *C. krusei*, *C. tropicalis* and *C. auris* often exhibit intrinsic or acquired resistance to typical antifungal agents. *C. glabrata* and *C. krusei* often show reduced susceptibility to azoles and require the use of echinocandins or higher doses of alternative agents, which may not always be feasible in critically ill or renally compromised patients [9,10].

Differences in virulence, tissue tropism and biofilm-forming ability among non-albicans species complicate both diagnosis and eradication, leading to persistent or recurrent infections despite antifungal treatment [9,10,11]. Of particular note is a relatively new species, *Candida auris*, which has become a global health concern. Since it was first identified in Japan (2009), it has spread rapidly to more than 35 countries [12,13,14]. The World Health Organization (WHO) has classified *C. auris* as a critical priority pathogen on its fungal priority list. The significance of *C. auris* lies in its high resistance to antifungal agents, which severely limits therapeutic options. In addition, the challenges associated with its laboratory identification, combined with its ease of transmission, provide ideal conditions for epidemic outbreaks. Furthermore, *C. auris* can persist in the environment for weeks, and the lack of effective decolonization methods makes controlling its spread a major challenge [13,15]. This shift is also correlated with variable and increased mortality rates of up to 40–60% [15]. Studies have shown that infections caused by non-candida species, especially *C. glabrata* and *C. auris*, are associated with delays in appropriate antifungal therapy, which is a critical factor, and higher 30-day mortality compared to *C. albicans* [16,17].

The distribution of *Candida* species varies significantly between regions, with direct implications for treatment strategies. In North America and Northern Europe, *C. glabrata* is more prevalent and, as we mentioned before, has reduced susceptibility to fluconazole, making echinocandins the preferred first-line therapy. In Spain, non-*albicans Candida* species are increasingly implicated in candidemia. A multicenter study (CANDIMAD) [18] from 2020 to 2022 reported a rise in *C. parapsilosis* from 21.6% to 36.1%, alongside a decrease in *C. albicans*. Notably, fluconazole-resistant *C. parapsilosis* strains, particularly the CP-451 genotype, have become endemic in several hospitals.

Latin America and parts of Asia report a higher prevalence of *C. parapsilosis*, which tends to be more susceptible to azoles but has higher minimum inhibitory concentrations (MICs) for echinocandins. Meanwhile, *C. tropicalis* is particularly common in Southeast Asia and India, often associated with hematological malignancies and high virulence [19].

Regarding IAC, Dupont H et al. analyzed samples from patients with peritonitis admitted to the ICU, of whom 30.6% had *Candida* spp. Peritonitis [20]. The most frequently isolated species in these cases was *C. albicans* (74%), followed by *C. glabrata* (17%). Vergidis P et al. [21] identified 163 IAC cases and 161 candidemias among more than 34,000 patients, with a rate of 4.7 episodes per 1000 admissions. A multicenter study (EUCANDICU) conducted in 23 ICUs across nine European countries analyzed 570 episodes of invasive candidiasis (7 episodes per 1000 ICU admissions). Of these cases, 65% were candidemia, 29% were IAC and 5% were IAC in combination with candidemia. A noteworthy finding of this study is that 43% of cases involved non-albicans *Candida* species, with an 8% rate of co-infection by two species [2].

As is evident, the epidemiology of both candidemia and IAC has shifted towards non-albicans *Candida* species. The reasons for this shift are thought to be linked to the increasing use of azoles for “prophylactic” purposes, as well as the rise in gastrointestinal pathology among older patients with more comorbidities. These findings should be carefully considered when determining the empirical treatment for these patients. The overall quality of the available evidence remains poor and uniform recommendations cannot be made.

## 3. Risk Factors

As outlined in various publications by different authors, there are different risk factors associated with the likelihood of invasive candidiasis [1,2,3,4,5,8]. There are those patient-related, treatment-related and procedural-related factors. Table 2 presents the most common risk factors. Prior colonization, especially multifocal colonization, is a crucial factor that should be assessed in critically ill patients, as it plays a key role in diagnosing or predicting IAC using various scoring systems. Multifocal colonization should be actively sought in all critical patients with an ICU stay of more than 7–10 days, especially if they have risk factors.

Another important factor is prior antibiotic treatment, which is considered a significant risk factor for IAC in nearly all studies. It is important to note that approximately 60% of ICU patients receive antibiotic treatment for various reasons [22], which significantly increases the number of patients at potential risk for IAC. Restrictive policies on the use of antimicrobials and a reduction in the duration of treatments are measures that could be taken to reduce the risk of IAC.

Finally, the widespread overuse of fluconazole for preventive or prophylactic purposes exerts selective pressure not only on *Candida* spp. but also markedly contributes to their resistance to azoles.

Research has identified comparable risk factors for IAC. Bassetti et al. published a study on predictive factors for IAC in ICU patients, using a logistic regression analysis from the EUCANDICU study [23]. The authors observed that recurrent gastrointestinal perforation (OR = 13.9, 95% CI 2.6–72.8), anastomotic leakage (OR = 6.6, 95% CI 1.9–21.9), intra-abdominal drains (OR = 6.5, 95% CI 1.7–25.0), receiving antifungal treatment (OR = 4.2, 95% CI 1.04–17.4) or antibiotic treatment for more than seven days (OR = 3.7, 95% CI 1.3–10.5) were independent predictors. As Bassetti et al. highlighted in another multicenter study involving more than 300 cases of candidemia, intra-abdominal infection increases the risk of developing shock (OR = 2.18, 95% CI 1.04–4.55) [24]. Dupont H et al. used a multivariate model to assess whether the presence of *Candida* spp. could be predicted in critically ill patients with peritonitis. The authors found that a high gastrointestinal origin (above the Treitz ligament) increased the risk of *Candida* isolation by 2.4 times (OR = 2.38), as did antibiotic administration (OR = 2.25) and the presence of shock (OR = 2.45) [20].

As antibiotic treatment is mandatory in patients with peritonitis, it remains a non-modifiable risk factor. However, it should be specifically considered when deciding whether to initiate antifungal therapy. Furthermore, as will be discussed later, in more than 40% of cases, bacterial and fungal infections coexist, and this association appears to have a significant impact on patient mortality [25,26].

Interactions between *C. albicans* and gut bacteria can be either antagonistic or synergistic. Species like *Pseudomonas aeruginosa*, *Salmonella* spp. and *Lactobacillus* exert inhibitory effects on *C. albicans*. Understanding these dynamics is crucial for developing targeted probiotic or microbiota-modulating therapies to prevent overgrowth and invasive intestinal candidiasis [27]. There are still many aspects of the pathogenesis of IAC that are not fully understood. One area of increasing interest is the role of gut enterotypes and patient response. These enterotypes are largely influenced by the host’s diet and shape the metabolic landscape of the gut microbiota. Enterotype I bacteria derive their energy mainly from carbohydrates through glycolysis and the pentose phosphate pathway. In contrast, enterotypes II and III harbor microbial populations capable of degrading glycoproteins present on the mucosal surface. This activity can weaken the epithelial barrier, promote immune evasion and create a microenvironment conducive to fungal colonization and overgrowth. Specifically, this alteration may predispose to the invasion of *Candida* spp. and contribute to the initiation or progression and lack of resolution of intra-abdominal infections [28]

## 4. Diagnosis

The isolation of *Candida* spp. in blood (candidemia) or other sterile body fluids is a clear criterion for infection. However, the diagnostic significance of its isolation in respiratory samples (which has no significance except in immunocompromised patients) or in samples obtained by puncture/surgery (especially those obtained from abdominal drains, which are potentially contaminated) remains a subject of controversy.

The initial diagnostic approach involves the identification of patients with the previously mentioned risk factors. Secondly, it is essential to obtain intra- or perioperative samples (fluid or tissue), as well as any other clinically relevant samples. Surveillance cultures also play an important role in diagnosis by demonstrating multiple colonization (Table 3). Additionally, predictive scores (see Table 4) can be applied to patients with more than two weeks of ICU stay, allowing us to assess the risk probability of invasive candidiasis.

Thirdly, the serological determinations of different biomarkers are also considered, although these are not available in many centers and their diagnostic capacity is still under discussion.

The gold standard for diagnosing invasive candidiasis is blood culture, although this method has significant limitations. Its sensitivity ranges from 50% to 75%, and the time required from sample collection to fungal identification is at least 48–72 h. This is particularly problematic because yeast replication is often slower than bacterial replication, leading to delayed blood culture positivity. Once a blood culture has returned a positive result, further identification is required [29,30].

**Table 3:** Different diagnostic techniques for invasive candidiasis.

Methods	Comments	Performance	Key Points
Detection of mannan antigen (M-Ag) and anti-mannan antibody (M-Ac)	Combined detection of antigen and antibody is recommended for all patients suspected of invasive candidiasis.	Se: 80–90% Sp: 85–90% PNV: 85–95% PPV: 70–85% [31]	Sensitivity varies depending on *Candida* species (e.g., lower for *C. krusei* or *C. parapsilosis*); limited utility in neutropenic patients or those with strong immunosuppression.
Detection and quantification of anti-mycelial or anti-germ tube antibodies (CAGTAs)	When antibody titers exceed 1:160. Additionally, it may help distinguish between transient or catheter-related candidemia (CAGTA–) and deep-seated infection-associated candidemia (CAGTA+).	Se: 85% Sp: 95% PPV: 72–89% NPV: 92–97% [32]	A negative CAGTA result has a high NPV, meaning it is especially useful to rule out invasive candidiasis in high-risk patients.
Determination of 1,3-ß-D-glucan	1,3-ß-D-glucan is a fungal cell wall component released during infection. It is a non-specific biomarker and does not identify the causative species.	Se: 70–90% Sp: 75–95% PPV: 50–80% (depends on pre-test probability). NPV: 95–100% in high-risk populations [33]	A negative BDG test is particularly valuable for ruling out invasive candidiasis (high NPV), especially in ICU and immunocompromised patients. It is valuable for discontinuing antifungal treatment.
In situ hybridization PNA-FISH Yeast Traffic Light	Uses species-specific peptide nucleic acid (PNA) probes that hybridize in situ with the target DNA of various *Candida* species, producing fluorescence in different colors. It also allows the identification of fluconazole-sensitive species, facilitating treatment adjustments and potential de-escalation.	Se: 92–100% Sp: 95–100%. PPV: >98% NPV: >98% [34]	Especially valuable where *C. glabrata* (often fluconazole-resistant) must be differentiated from *C. albicans* early. Rapid technique.
Mass spectrometry with soft ionization (MALDI-TOF)	This technique is performed on a positive blood culture. It identifies bacteria and fungi through proteomic analysis, allowing accurate identification of fungal isolates in solid culture media in less than 30 min.	Se: 95–100% Sp: 98% PPV: 95% NPV 95% [35]	Highly useful for early targeted therapy when paired with automated blood culture systems.
Detection of nucleic acids via polymerase chain reaction (PCR)	This technique enables the detection of bacterial DNA and certain *Candida* species in blood or sterile fluids using real-time multiplex PCR or microarrays. The limitation is that it requires a positive blood culture, but it allows rapid detection (1 h).	Se: 80–95% Sp: 90–98% PPV: up to 90% NPV: 95% [33]	Early and direct detection of *Candida* DNA from blood or sterile fluids, often before cultures become positive. Particularly useful in high-risk populations (e.g., ICU, neutropenia and post-surgery). False positives may occur due to detection of colonization or non-viable DNA.
T2 Rm (Candida panel)	This technique detects *Candida* spp. using magnetic resonance within 3–5 h.	Se: 91–95% Sp: 98–99% PPV: 70–80%% NPV: >99% [36]	Allows species-level diagnosis within hours—much faster than cultures (48–72 h). Useful in patients with suspected candidemia, but negative cultures can reduce empirical antifungal use and improve targeted therapy. Unfortunately, this device has been withdrawn from the market in Europe.

Sensitivity (Se); Specificity (Sp); Positive predictive value (PPV); Negative predictive value (NPV).

### 4.1. Biomarkers for Diagnosing Invasive Candidiasis

León C et al. evaluated the usefulness of different biomarkers in critically ill patients with intra-abdominal pathology, both individually and in combination. The study’s key findings included the positive results for 1,3-ß-D-glucan (BDG) and anti-mycelial or anti-germ tube antibodies (CAGTAs) in a single sample, or at least one of them in two consecutive samples, which was identified as the most effective strategy for diagnosing invasive candidiasis (IC). In contrast, the individual or combined positivity of C-PCR, mannan antigen (M-Ag) and anti-mannan antibody (M-Ac) was found to have minimal value in distinguishing IC from colonization. The authors concluded that BDG is the most suitable biomarker for diagnosing IC in critically ill patients with acute abdominal complications. Its diagnostic performance is enhanced when combined with CAGTAs [37]. However, it should be noted that other studies have not consistently replicated these findings.

Macrophage-derived CD36+ exosomes have emerged as promising non-invasive biomarkers for Candida albicans infection, showing specificity and elevated levels during fungal exposure. Their unique immunomodulatory content and detectability in biological fluids suggest diagnostic potential for early infection detection and monitoring, surpassing the limitations of conventional diagnostic methods [38].

In our opinion, and in agreement with the majority of researchers, BDG is the biomarker that should be requested in clinical practice, when available, in patients at high risk of invasive candidiasis. Although antifungal treatment should be initiated empirically and early in critically ill and unstable patients with risk factors for candidemia, without waiting for biomarker results, the determination of BDG may contribute to the diagnosis and possibly to the continuation or withdrawal of treatment, given its high negative predictive value.

### 4.2. Predictive Scores for Invasive Candidiasis

Several researchers have attempted to develop the most effective predictive scores to assess the risk of invasive candidiasis in patients with risk factors. Table 4 provides a comprehensive overview of the main predictive scores, their characteristics and limitations. One of the earliest predictive scales was the Pittet colonization index [39], which was designed for surgical patients. While it is a useful tool, it requires quantitative cultures, which can put a strain on microbiology services. This study demonstrated that a corrected colonization index greater than 0.4 had up to a 100% positive and negative predictive value. León C et al. conducted a prospective study involving 1720 critically ill patients with an ICU stay of over 7 days, leading to the development of the “*Candida* score” or “Sevilla Score”. A score greater than 2.5 was able to predict *Candida* infection with 81% sensitivity and 74% specificity [40]. This score was validated in a subsequent prospective study of 1107 critically ill, non-neutropenic patients, confirming that a *Candida* score ≥3 was useful for distinguishing infection. Its diagnostic accuracy was superior to the Pittet colonization index. It is important to highlight that prior colonization by *Candida* spp. is a mandatory criterion for a *Candida* score > 3 [41].

As discussed later, different authors have implemented these scales to enable early treatment, observing a decrease in the incidence of invasive candidiasis, though often not significant, with no impact on mortality. In our opinion, patients with a prolonged stay in the ICU, risk factors and a *Candida* score > 3 should be considered highly suspicious for invasive candidiasis (provided there is multifocal colonization) and should be started on antifungal treatment early. However, other circumstances that may increase the risk of fungal infection should be considered. In particular, patients with decompensated cirrhosis, hepatitis and those who have undergone liver transplantation are at high risk of developing *Candida* infections. Many factors, such as host immune dysfunction, blood–brain barrier dysfunction, malnutrition and microbiome alterations, contribute to this increased risk of developing fungal infections.

In these patients with a high pretest probability of invasive candidiasis, and where available, it would be appropriate to use molecular diagnostic techniques or biomarker determination (BDG) to refine the diagnosis and avoid overuse of antifungals.

**Table 4:** Predictive scales for invasive candidiasis used in clinical practice.

Scale	Variables	Cutoff Point	Sensitivity/Specificity	Comments
Pittet Colonization Index [39]	Ratio of the number of colonized sites to the number of cultured sites	≥0.5	100%/69%	In surgical patients, it requires quantitative cultures from all sites, which increases cost and microbiology processing time.
Corrected Pittet Colonization Index [39]	Ratio of the number of sites with high colonization (>10^5^ CFU) to the number of cultured sites	≥0.4	100%/100%	Similar to the previous index.
*Candida* Score [40]	Multifocal colonization (1 point) Abdominal surgery (1 point) Parenteral nutrition (1 point) Severe sepsis—shock (2 points)	≥3	77%/66%	More practical for ICU patients with >7 days of stay. Note that with the cutoff point of 3, multifocal colonization is mandatory.
Nebraska Rule [42]	Stay > 4 days in ICU + Broad-spectrum antibiotics Presence of CVC Abdominal surgery Steroid treatment Parenteral nutrition Average stay before ICU	2.45	84%/60%	More complex to implement as it requires performing a regression calculation with coefficients of the different risk factors.

## 5. Mortality

The mortality rate for invasive candidiasis is high, ranging from 40% to 60% according to various authors [1,2,3,4,5,8,43]. The mortality rate for transient candidemia (e.g., secondary to catheter use) is significantly lower than that for candidemia caused by a deep focus (e.g., endocarditis and intra-abdominal infections). Researchers have explored the impact of early antifungal treatment (see below) and have not observed a favorable effect on mortality in patients with invasive candidiasis. It is crucial to consider three key factors in the pathogenesis of this condition to understand its high mortality.

Firstly, a high fungal load may be a result of broad-spectrum antibiotic use, especially anti-anaerobic antimicrobials. This elevated load or colonization facilitates the translocation of the yeast from the gastrointestinal tract in patients who commonly suffer from prolonged ileus and severe intestinal microcirculation alterations.

Secondly, the disruption of the integrity of both the cutaneous–mucosal barrier (candidemia) and the gastrointestinal tract (IAC) must be considered, whether due to spontaneous perforations or prolonged use of drains or chronic inflammatory processes (e.g., pancreatitis).

Finally, the third element is the dysfunction of immunity that every critically ill, non-neutropenic patient experiences, leading to dissemination to deep tissues [44]. In this context, it is important to note that tertiary peritonitis has a poor prognosis beyond treatment due to immune dysfunction in the peritoneum, which hinders the resolution of the infectious process.

Despite acknowledging these factors, the positive impact of appropriate antifungal treatment is indisputable. In the initial study by Morrel L et al., early treatment (within 12 h) was associated with a significant reduction in mortality (12%) compared to late treatment (>12 h), which had a 30% mortality rate in patients with candidemia [44]. This finding has been replicated by other researchers [45,46]. In their multivariate analysis, Morrel L et al. [36] found that treatment delay was associated with twice the risk of death (OR = 2.06). In the same study, prior antibiotic administration was found to be associated with a higher mortality (OR = 4.06). Garey K et al. [40] found that fluconazole treatment in patients with candidemia was associated with lower mortality (15%) when administered on day 1 (mortality 23%), day 2 (mortality 37%) or day 3 (mortality 42%) after blood culture. These authors reported a 1.5 higher risk of mortality (OR = 1.52) for each day of treatment delay [46]. Finally, Zilberberg MD et al. reported a higher mortality rate (28.9%, *p* = 0.056) in patients with candidemia who received inappropriate treatment. It is noteworthy that in this study mortality was 0% in patients with appropriate treatment [47].

In an observational, prospective, multicenter study in critically ill patients with community-acquired intra-abdominal infection, the isolation of *Candida* spp. in peritoneal samples was closely associated with mortality [48]. Despite the introduction of new antifungals, such as echinocandins in the early 2000s, mortality in IAC remains above 50%. The shift in IAC etiology towards non-albicans species is partly responsible for this high mortality, along with increased empirical inappropriate treatment due to high resistance to commonly used antifungals. Of particular concern is the emergence of *C. auris*, the first fungal pathogen classified by the Centers for Disease Control and Prevention (CDC) as an urgent public health threat due to its association with extremely high mortality rates and the potential to develop resistance to all current antifungals.

Shorr A et al. [49] and Vardakas KZ et al. [50] conducted two meta-analyses of randomized clinical trials assessing prophylaxis with azoles in surgical ICU patients. These analyses concluded that there was a reduction in the incidence of fungal infections, although there was no impact on mortality. Conversely, a meta-analysis encompassing 12 studies conducted on non-neutropenic, critically ill patients determined that azole prophylaxis led to a reduction in mortality (RR, 0.76; 95% CI, 0.59–0.97) and the incidence of invasive fungal infection (RR, 0.46; 95% CI, 0.31–0.68) [49,50].

At this stage in the review, we may feel uncertain, given the observed association between the administration of antibiotics and the presence of *Candida* spp., as well as the previously documented link between prior antibiotic use and higher mortality, particularly in cases of IAC. Consequently, the question arises as to whether it is advisable to administer antibiotics to patients with peritonitis. The answer is clear: in peritonitis, antibiotics should be administered, but we must bear in mind that this creates a predisposing condition for an added or coexisting fungal infection. Consequently, we will undertake an active search for fungal infection in these patients, utilizing the available resources to initiate treatment promptly. However, we must also consider that a strategy of generalized early treatment can lead to the excessive use of antifungal medication and a possible increase in resistance to azoles or to infections caused by non-albicans species.

## 6. Treatment

The management of complicated intra-abdominal infection is a complex scenario where other conditions, beyond antifungal treatment, play a significant role. The patient’s specific conditions, as well as early and proper control of the source of infection, are key factors in determining the outcome for these patients. Once the source of infection has been addressed, the role of antifungal therapy becomes crucial.

Several authors have used different definitions for treatments related to possible or probable *Candida* spp. infection in their studies. This variability complicates comparisons and limits the strength of conclusions. Therefore, in this review, we will apply the definitions presented in the 2019 guidelines of the European Society of Intensive Care Medicine/European Society of Infections working group [51]:

(1) “Prophylaxis” is antifungal treatment given without clear evidence of infection to critically ill patients at high risk of invasive candidiasis due to intrinsic factors (e.g., immunosuppression) or hospitalization-related factors (e.g., septic shock, abdominal surgery, prolonged ICU stay or broad-spectrum antibiotics);
(2) “Pre-emptive therapy” is antifungal treatment of critically ill patients at high risk of invasive candidiasis guided by fungal biomarkers or risk assessment scores associated with multifocal colonization;
(3) “Empirical therapy” is antifungal treatment of critically ill patients with signs and symptoms of infection (e.g., persistent fever of unknown origin) who have specific risk factors for invasive candidiasis, regardless of biomarker results;
(4) “Targeted therapy” is antifungal treatment based on microbiological findings confirming the presence of *Candida* spp. and its susceptibility to different antifungal agents.

To ensure optimal treatment efficacy and minimize the risk of re-resistance, it is essential that the antifungal agent reach adequate concentrations at the site of infection, which in this case could be intraperitoneal. It is important to emphasize that there is limited and often conflicting information on intra-abdominal antifungal concentrations in critically ill patients with peritonitis [52]. It is important to recognize the limitations of extrapolating data from blood concentrations to intraperitoneal levels. The central compartment, which includes the bloodstream, provides a convenient method for monitoring drug levels and assessing pharmacokinetic/pharmacodynamic (PK/PD) behavior to optimize drug efficacy.

However, it is well established that patients in critical condition exhibit significant variability in drug PK behavior due to several factors. These include a marked increase in drug distribution volume and elevated renal clearance, particularly during the initial phases of critical illness [53]. These PK alterations primarily impact hydrophilic drugs, such as echinocandins, which are the first-line treatment recommended by various international guidelines. If the dosage of these drugs is not properly adjusted, often requiring dose escalation or loading doses, the resulting drug concentrations may fall below the levels needed to act against pathogens with reduced susceptibility, thus promoting the emergence of so-called “resistant mutants” [54]. In cases of intra-abdominal infections, the diffusion of drugs into the peritoneal cavity is further hindered by poor penetration, as well as the presence of surgical drains (often high-output or frequently flushed). Additional factors that can impact the ability to achieve adequate drug concentrations include high body weight (over 100 kg), low albumin levels common among critically ill patients and the frequent use of intermittent or continuous renal replacement therapies [55].

It is essential to consider all these factors when selecting the most appropriate antifungal treatment for each patient’s specific condition.

Three classes of drugs (azoles, echinocandins and polyenes) make up the available therapeutic arsenal for treating candidiasis and IAC. As previously mentioned, *Candida albicans* remains the most frequently isolated species. However, the frequent presence of non-albicans species and the emergence of resistance to azoles and echinocandins make empirical treatment a significant challenge that must be addressed on a daily basis (see Table 5).

### 6.1. Azoles

Azoles are a class of antifungal agents that disrupt ergosterol biosynthesis in the fungal membrane by inhibiting 14-α-sterol demethylase, a microsomal cytochrome (CYP). These compounds are categorized into two main groups: imidazoles (e.g., clotrimazole, miconazole and ketoconazole) and triazoles (e.g., fluconazole, voriconazole and isavuconazole). Systemic triazoles are metabolized more slowly and have fewer effects on human sterol synthesis compared to imidazoles. All azoles undergo hepatic metabolism through different cytochrome P450 (CYP) enzymes, albeit with varying intensities, leading to numerous drug interactions that must be carefully considered.

#### 6.1.1. Fluconazole

The primary mechanism of action of fluconazole consists of the inhibition of fungal cytochrome P-450 by blocking 14-alpha-lanosterol demethylation, a key step in the biosynthesis of fungal ergosterol, and a moderate inhibitor of CYP3A4. A direct relationship has been observed between the dose, the MIC (minimum inhibitory concentration) and clinical efficacy.

In addition to multiple observed drug interactions, there is a risk of increasing plasma concentrations of other compounds metabolized by CYP2C9, CYP2C19 and CYP3A4 when co-administered with fluconazole (such as anticoagulants, antidepressants, benzodiazepines, carbamazepine, calcium channel blockers, fentanyl, sirolimus, tacrolimus, losartan, phenytoin, prednisone, etc.). Due to its long half-life, fluconazole continues to inhibit enzymes for four to five days after discontinuation [56]. Fluconazole can be administered orally (with good bioavailability, though not widely recommended in critically ill patients) or intravenously. It is important to note that food intake does not affect oral absorption. Peak plasma concentrations (Cmax) are reached between 0.5 and 1.5 h post-dose and are proportional to the administered dose. Steady-state levels are achieved within 4 to 5 days after multiple once-daily doses. A loading dose of 200 mg on day 1, which is twice the usual daily dose, increases plasma levels to 90% of steady-state concentrations by day 2, making this a recommended strategy for critically ill patients. The volume of distribution (VD) is 40 L, with low protein binding (10%). This means that while a large proportion of the drug remains free to exert its action, its renal elimination is significantly increased in hyper-filtering patients or those undergoing renal replacement therapies, requiring reconsideration of its use in such patients. Fluconazole distributes widely, achieving high concentrations in cerebrospinal fluid (CSF), the lungs, skin, the cornea and urine. The recommended loading dose is 800 mg (400 mg every 12 h) on the first day, followed by 400 mg/day. Maintenance doses may vary depending on patient conditions [56,57].

#### 6.1.2. Voriconazole

The primary mechanism of action of voriconazole is the inhibition of 14-alpha-lanosterol demethylation, which is mediated by fungal cytochrome P-450. This is a key step in the process of fungal ergosterol biosynthesis. The accumulation of 14-alpha-methyl sterols is associated with the subsequent loss of ergosterol in the fungal cell membrane [58]. Voriconazole can be administered orally (though rarely in critically ill patients) or intravenously. Its pharmacokinetics are non-linear due to metabolic saturation, meaning that a disproportionate rise in plasma concentrations is seen with increases in dose. Administering a loading dose helps to achieve plasma concentrations close to the steady state within the first 24 h, making this a particularly important strategy for critically ill patients. The oral bioavailability of voriconazole is 96%; however, it is influenced by CYP3A4 activity and a transporter protein, as well as by high-fat meals, which reduce Cmax and AUC (area under the curve). Voriconazole distributes widely, with a volume of distribution of 4.6 L/kg. Voriconazole has a plasma protein binding of 58%, and it reaches effective concentrations in the CSF, lungs, brain, liver, kidneys and spleen. As previously mentioned, voriconazole is metabolized by, and also inhibits, cytochrome P450 enzymes CYP2C19, CYP2C9 and CYP3A4. The presence of inhibitors or inducers of these isoenzymes can result in alterations to voriconazole plasma concentrations. Voriconazole can also increase plasma concentrations of substances metabolized through these CYP450 enzymes, particularly those processed by CYP3A4, as it is a potent inhibitor of this enzyme.

This means that voriconazole exhibits significant interindividual variability, requiring plasma drug level monitoring to ensure therapeutic effectiveness. Genetic polymorphisms in CYP enzymes (such as those found in 15–20% of the Asian population) slow drug elimination, increasing the risk of toxicity. While these polymorphisms are less prevalent in Caucasian populations (with a frequency of less than 5%), some patients exhibit rapid metabolization, which can complicate the achievement of adequate drug levels [57].

#### 6.1.3. Isavuconazole

Isavuconazole is a fungicide that exerts its effects by blocking ergosterol synthesis, a key component of the fungal cell membrane. This is achieved through the inhibition of cytochrome P450-dependent lanosterol 14-alpha-demethylase. This results in an accumulation of methylated sterol precursors and a reduction in ergosterol within the fungal membrane, weakening its structure and function.

Isavuconazole is administered as a prodrug that is rapidly hydrolyzed in plasma to its active form. It can be administered orally (with high bioavailability but rarely used in critically ill patients) or intravenously. Its pharmacokinetics are linear, with Cmax and AUC values proportional to the administered dose, and drug accumulation occurs with multiple dosing. Isavuconazole distributes extensively, with a high VD (>400 L). It has strong protein binding (>99%) and achieves adequate concentrations in CSF, the lungs, ascitic fluid and the eye. The drug is metabolized in the liver via CYP3A4, CYP3A5 and uridine diphosphate glucuronosyltransferase (UGT). While it interacts with various drugs, isavuconazole has a better safety profile than voriconazole. The drug is slowly eliminated from the body, with a half-life of 84–117 h, likely due to its high protein binding and large distribution volume. Reduced susceptibility to triazole antifungals has been associated with mutations in the fungal CYP51A and CYP51B genes, which encode the 14-alpha-demethylase enzyme involved in ergosterol biosynthesis.

Fungal strains with in vitro susceptibility to isavuconazole have been reported, but cross-resistance with voriconazole and other triazole antifungals cannot be ruled out. The recommended regimen for isavuconazole is a loading dose of 200 mg every 8 h for the first 48 h (total of 6 doses), followed by a maintenance dose of 200 mg once daily, starting 12–24 h after the last loading dose. As isavuconazole does not require therapeutic drug monitoring, it is considered the most appropriate option for critically ill patients [57,59].

### 6.2. Echinocandins

#### 6.2.1. Anidulafungin

Anidulafungin is a semi-synthetic echinocandin, a lipopeptide derived from a fermentation product of Aspergillus nidulans that is administered intravenously. It selectively inhibits 1,3-β-D-glucan synthase, preventing the formation of 1,3-β-D-glucan, an essential component of the fungal cell wall [60]. A very low interindividual variability in systemic exposure has been observed, and a steady state is reached the day after the loading dose is administered. The half-life is 24 h, with a volume of distribution (Vd) of 30–50 L. Anidulafungin extensively binds (>99%) to human plasma proteins. While it distributes well in the lungs, no data are available on its penetration into the cerebrospinal fluid (CSF). Its antifungal action is concentration-dependent, meaning it is associated with AUC/MIC and Cmax/MIC targets, making it crucial to ensure an adequate dose. The standard dose has been associated with a lower AUC0-24 in critically ill patients compared to healthy volunteers. Due to its extensive protein binding, only the free fraction is considered capable of diffusing into the peritoneum, potentially compromising its efficacy. While different studies suggest that peritoneal concentrations would meet the classical efficacy targets defined by an AUC/MIC of 3000 for all *Candida* species, a recent study by Gioia F et al. [60] showed that the AUC/3000 results in serum were 0.042, 0.032 and 0.022 for anidulafungin, micafungin and caspofungin, respectively. These levels would be adequate for the treatment of *C. albicans* (0.03 mg/L) but suboptimal for *C. glabrata*, *C. krusei* and *C. tropicalis* (0.06 mg/L), potentially creating an environment conducive to resistance development.

It has been hypothesized by several authors that IAC may act as a hidden reservoir for echinocandin-resistant *C. glabrata* species. *C. glabrata*, which is increasingly present in IAC, exhibits a significant and varied adaptive response to its environment, linked to intrinsic mutations in FKS genes, which encode β-1,3-D-glucan synthase. These mutations result in an increased chitin content in the cell wall and paradoxical growth when high doses of echinocandins are administered. Finally, concentrations <2 mg/L lead to the selection of resistant mutants, which emerged rapidly (<48 h) in laboratory studies at different echinocandin concentrations. Metabolism occurs in the plasma, with no evidence of hepatic metabolism, making anidulafungin a drug with few interactions. It undergoes slow chemical degradation at physiological temperature and pH, resulting in an open-ring peptide that lacks antifungal activity. Renal elimination is minimal (<10%). A single 200 mg loading dose should be administered on day 1, followed by a daily maintenance dose of 100 mg [57,60,61,62].

#### 6.2.2. Rezafungin

Rezafungin is a novel echinocandin that selectively inhibits fungal 1,3-β-D-glucan synthase with inhibition of fungal cell wall synthesis, resulting in rapid and concentration-dependent fungicidal activity in Candida species. It has state-of-the-art pharmacokinetics with remarkable stability, high solubility and an exceptionally long half-life, allowing early exposure to high drug levels. It can therefore be administered intravenously once a week, with a loading dose of 400 mg on day 1, followed by 200 mg on day 8 and once a week thereafter [63]. The pharmacokinetic results in the single ascending dose study were consistent with the post-first-dose pharmacokinetic results in the multiple ascending dose study for each dose cohort. At all doses investigated in the single ascending dose study, mean maximum plasma concentration (Cmax) values ranged from 2.76 to 22.7 g/mL, and the values corresponding to the mean area under the concentration–time curve from time zero to 168 h (AUC0-168) ranged from 145 to 1160 g-h/mL. Both Cmax and AUC increased in a dose-proportional manner, and mean half-life (t1/2) values of 80 h were observed during the first week (up to day 7) of plasma sampling (a longer terminal t1/2 of 125 to 146 h was calculated by incorporating data from later sampling times [days 14 and 21]) [64].

Rezafungin is rapidly distributed with a volume of distribution similar to that of body water (≈40 L) and binds to 97% of plasma proteins. Like the other echinocandins, it is not affected by intermittent/continuous renal replacement techniques. Furthermore, it undergoes little or no biotransformation and is mainly excreted in the feces, with less than 1% of the drug being excreted in the urine, with an average plasma half-life of 127 to 146 h [65].

Rezafungin has a broad spectrum of activity against *Candida* and *Aspergillus* species, including *Candida auris* and subsets of fungal strains resistant to other antifungals (echinocandins and azoles), and has demonstrated therapeutic and prophylactic efficacy in animal models of candidiasis, aspergillosis and Pneumocystis pneumonia. Rezafungin has demonstrated safety and tolerability comparable to current echinocandins. In addition, rezafungin has demonstrated chemical and metabolic stability and non-hepatotoxicity compared to anidulafungin [61,62,63], with increased biofilm activity.

In phase III trials, rezafungin has demonstrated non-inferiority to caspofungin for the treatment of invasive candidiasis (ReSTORE trial) [66]. In addition to its weekly dosing, rezafungin offers other potential pharmacokinetic and pharmacodynamic advantages, such as excellent penetration into anatomically challenging sites, such as intra-abdominal collections, and a potentially lower risk of promoting local resistance. This positions it as a promising option for deep infections, particularly intra-abdominal infections, justifying its commercialization and inclusion as a first-line agent in recent guidelines for the treatment of candidiasis [67].

### 6.3. Polyenes

#### Amphotericin B (AMB)

Liposomal amphotericin B (L-AMB) is a heptane-derived macrolide that contains seven conjugated trans double bonds and 3-amino-3-6-dideoxy-mannose (mycosamine) linked to the main ring via a glycosidic bond. The amphoteric behavior that gives the drug its name is due to the presence of a carboxyl group on the main ring and a primary amino group on mycosamine, which confer aqueous solubility at pH extremes. Its effectiveness as a fungicide or fungistatic depends on the achieved concentration and the specific fungal species. Its mechanism of action involves binding to ergosterol in the fungal membrane, leading to membrane disruption and increased permeability. Its antifungal activity is concentration-dependent, meaning it is related to the plasma concentration (Cmax) over the minimum inhibitory concentration (MIC), demonstrating a prolonged post-antifungal effect [68,69].

L-AMB achieves higher plasma concentrations (Cmax) than AMB and has a longer exposure time (AUC0-24). Following administration, L-AMB reaches the steady state rapidly, generally within the first four days of the same dose. A key feature of L-AMB is its action against biofilm formation, which is directly implicated in IAC [68,70,71]. The pharmacokinetics of L-AMB are largely dependent on the composition and size of the liposomal or lipid complex particles. Large structures, such as the lipid complex (Abelcet), are rapidly absorbed by the mononuclear phagocyte system, whereas smaller liposomes remain in the circulation for extended periods.

The main limitations to the use of AMB are nephrotoxicity and hepatotoxicity. However, AMB has been withdrawn from the market in many European countries, and lipid formulations, especially liposomal (L-AMB), are the most widely used. AMB molecules are stabilized by phospholipids and cholesterol within the liposomal bilayer, which reduces toxicity to animal cells. The small size of the liposomal particles (100 nm or less) ensures that drug levels remain high in the bloodstream, reducing distribution to organs, including the kidneys, and contributing to improved safety and reduced nephrotoxicity [62,63,64,65]. Studies by our group with real-world data have confirmed the adequate safety profile of L-AMB in critically ill patients [72].

The enhanced vascular permeability in critically ill patients has been shown to increase the transfer of L-AMB from the circulation to the lesions, thereby boosting its antifungal activity. In plasma, AMB remains associated with liposomes (97% at 4 h, 55% at 168 h) after L-AMB administration. It is assumed that L-AMB does not directly bind to target cells but instead facilitates AMB transfer from the liposomal membrane to the fungal cell membrane, which is critical for antifungal activity. The usual dose is 3–5 mg/kg/day, achieving adequate concentrations in the lungs, ascitic fluid and muscle tissue. While the drug does not penetrate the blood–brain barrier (BBB) efficiently, it is indicated for use in cases of inflammation, potentially in combination with other antifungals. Finally, due to its pharmacokinetics, plasma level monitoring is not required, as its efficacy does not correlate with observed blood levels [68,69,70,71].

## 7. Antifungal Therapeutic Drug Monitoring (TDM) Requirements

The importance of therapeutic drug monitoring (TDM) of antifungal agents in a variety of clinical settings is increasingly being recognized. However, there are no definitive data from large-scale clinical trials to support its use in all clinical settings (and there probably never will be). Most of the evidence supporting TDM is circumstantial. In addition, antifungal TDM can be costly and time-consuming, and its ultimate impact on clinical care may be difficult to estimate. Therefore, there is a broad consensus that TDM should not be used routinely (as is the case for some antimicrobials, such as aminoglycosides) but more selectively.

Beyond its indication, TDM is not available in most hospitals. Furthermore, TDM needs to have an adequate response time for results if treatment is to be adjusted early. Finally, most authors indicate that TDM should be determined when the antifungal is in the stable phase, which is achieved between 3 and 5 days. Detection of suboptimal levels after 72–96 h may not be useful in critically ill patients [73,74,75].

At this time, there is no evidence or indication to support the routine use of TDM for L-AMB, echinocandins, fluconazole and isavuconazole. Meanwhile, most authors assume that TDM is necessary for voriconazole [73,74,75,76,77]. However, some considerations must be taken into account.

For L-AMB, the structure of the liposomes deserves special attention. As with other liposomal formulations, drugs sequestered within this particle cannot reach diffusion equilibrium with the extravascular compartment. In addition, amphotericin B (AMB) released from a liposome binds strongly to plasma proteins (>90%, depending largely on the clinical status of the patient), and this aspect may affect the final exposure of AMB in the blood. Therefore, total AMB measured in the blood after administration of L-AMB may not be indicative of true exposure, and the clinical utility of monitoring L-AMB blood concentrations may be questionable since plasma levels are not related to clinical efficacy [75,76,77].

In general, it is not necessary to monitor fluconazole levels due to its favorable pharmacokinetic characteristics with rapid absorption and high bioavailability, prolonged distribution in the body and relatively high plasma levels. In addition, there is a direct correlation between the dose of fluconazole and the plasma levels achieved, which gives the drug predictable pharmacokinetics, and therefore it is not considered necessary to monitor plasma levels. However, it may be necessary to monitor plasma levels of fluconazole in certain groups of patients, in particular, patients on continuous renal replacement therapy (CRRT) or intermittent renal replacement therapy (IRRT). Although increasing the dose (800–1200 mg/day) may be an option to consider in this subgroup of patients, we believe that patients requiring CRRT/RRIT should be treated with therapy-unaffected antifungals such as L-AMB or echinocandins [73,74,75,76,77].

Echinocandins show a significant post-antifungal effect and therefore concentration-dependent activity. The Cmax/MIC and AUC0_24/MIC ratios (measured as total drug concentrations) are considered relevant pharmacodynamic indices for these drugs. Most experts believe that the data on the relationship between blood concentrations of echinocandins and therapeutic outcome are insufficient to support the routine use of DMT for these agents. However, factors such as obesity, age and clinical status may influence exposure and contribute to significant pharmacokinetic differences between these drugs. Although this PK/PD is described as a guarantee of efficacy for the treatment of *Candida* spp. infections and the levels far exceed the MIC90 for common *Candida* spp. strains, they would be insufficient for the treatment of *C. parapsilosis* or *C. glabrata* in intra-abdominal infections. Therefore, if TDM is not possible, we believe that loading doses and high doses of echinocandins should be used in critically ill patients [73,74,75,76,77].

Isavuconazole has linear and dose-proportional pharmacokinetics, which is useful for predicting blood concentrations in humans. It also has lower rates of adverse events and a better safety profile than other triazoles, with a lower propensity for cytochrome P450-mediated drug–drug interactions than voriconazole. Most experts agree that TDM is not necessary in patients receiving isavuconazole [73,74,75,76,77].

Most researchers maintain that TDM should be performed routinely in most patients receiving voriconazole. This azole has highly variable intra- and interindividual pharmacokinetics, attributed to different factors, such as pharmacogenetic polymorphisms, drug interactions (remember that it is mainly metabolized by CYP2C19, 2C9 and 3A4), alterations in gastrointestinal absorption, and even inflammation and the severity of the patient’s condition, as well as body weight. In our opinion, if TDM is not possible, voriconazole is not a treatment option for critical patients [73,74,75,76,77].

## 8. Combination Therapy Requirements

In general, the antifungal combination is not frequently used for invasive candidiasis or IAC, except possibly for *C. auris* because of its high level of resistance [78]. The international guidelines for the management of candidiasis generally do not contemplate a combination therapy except in some clinical circumstances such as CNS infections and endocarditis in which 5 flucytosine (5-FC) can be added to AMB [78,79,80].

Ideally, the overall goal of combination antifungal therapy is to achieve increased clinical efficacy of the antifungal agent and avoid toxicity to the patient. There are several advantages and reasons to consider a combination of drugs: (i) potential increased potency and extent of fungal killing (synergy); (ii) a broader spectrum of activity targeting potentially resistant pathogens; (iii) prevention of microbial resistance; and (iv) combination therapy may allow reduced doses of individual antifungal drugs, which may minimize toxicities.

It is equally important to consider potential antagonistic mechanisms, particularly with regard to the interaction between azoles and amphotericin B. Azoles bind avidly to ergosterol, causing its depletion in cytoplasmic membranes. Therefore, in combination with AMB, there are fewer binding sites available for AMB, resulting in a decrease in AMB binding and antifungal activity. However, the intensity of this interaction depends on the PK characteristics of the azoles. For example, highly lipophilic azoles (itraconazole) that accumulate in the cell membrane reduce the effect of AMB. On the other hand, hydrophilic azoles (fluconazole) do not accumulate in the membrane and therefore do not show antagonism [77]. In addition, there may be another type of antagonism caused by pre-exposure to azoles, which causes changes in the ergosterol in the membrane and reduces the effectiveness of AMB. In addition, there may be another type of antagonism caused by pre-exposure to azoles, which causes changes in the ergosterol in the membrane and reduces the effectiveness of AMB [78,79,80].

Although the combination of echinocandins with fluconazole has achieved a good response, no differences have been shown with respect to treatment with fluconazole alone. On the other hand, the combination of AMB with echinocandins exhibits erratic behavior ranging from a few cases of synergism to no action being evidenced or antagonism appearing in the majority of cases [79,80].

5-Flucytosine (5-FC) has a unique mechanism of action that theoretically makes it an ideal add-on drug for use in combination therapies, as it acts on RNA and prevents protein synthesis in fungi. It also inhibits DNA synthesis but is inactive against bacteria and human cells. Most in vitro studies have used this drug in combination with AMB, followed by combinations with azoles and echinocandins. Several studies have been conducted with difficult-to-treat *Candida* species. In a recent study of two-drug combinations against multidrug-resistant C. auris, 5-FC at 1.0 mg/L was found to improve most combinations with AMB, echinocandins and voriconazole [81].

However, except in specific situations (endocarditis or central nervous system infection), its administration in critically ill patients is not common because 5-FC has a well-known side-effect profile that includes both dose-related pharmacological toxicity and idiosyncratic pharmacological toxicity, including bone marrow toxicity (leukopenia and thrombocytopenia), hepatotoxicity, gastrointestinal intolerance and renal impairment, which generally requires TDM of this agent [81].

### Development of New Antifungal Agents

Based on the above, research continues to identify new antifungal targets and to develop specific drugs against these targets and to prevent resistance. Studies on antifungal drug resistance have identified several effective strategies such as increasing membrane β-glucan, improving tolerance through cellular stress, inhibiting biofilm formation and suppressing ergosterol biosynthesis (ERG). In addition, targets related to enzymes (acetyltransferases and deacetylases), fungal aspartate pathways, HSP90, CYP51, HDAC, SE, fructose bisphosphate aldolase (FBA), arachidonic acid pathways and sulphite transporters have gained in importance [82,83,84].

Several single-target drugs, such as suba-itraconazole, VT-1129, VT-1161 and VT-1598, and cell wall targeting drugs, such as amphotericin B coheates (CAmB), ibrexafungerp and fosmanogepix, as well as drugs targeting intracellular targets, including VL-2397, T-2307, MGCD290 and olorofim, are being studied intensively [76,77,78]. However, toxicity remains an issue, and to overcome these limitations, many research groups have focused on molecular hybridization to create multitarget drugs rather than traditional drug combinations. This shift has led to the design of multitarget ligands that are capable of acting simultaneously on multiple sites essential to the fungal life cycle with a single molecule, producing synergistic effects. This new approach could overcome challenges such as the development of resistance, limited pharmacokinetics and poor compliance associated with single-target drugs. The development of new drugs is of paramount importance for the future of patients with invasive fungal infections, but their detailed development is beyond the scope of this review.

## 9. When and How to Treat? The Role of Guidelines

The first question to consider is *When should I start empirical treatment for my patient?* As previously discussed, the decision to initiate prophylactic, anticipatory or empirical treatment will depend on the clinical conditions and risk factors of the patient. While most authors advocate against the routine use of antifungal prophylaxis in critically ill patients, we generally support the initiation of prophylactic treatment in patients with peritonitis and perforation of the upper gastrointestinal tract (above the Treitz angle), particularly in patients who use antacids and have gastric neoplasms. Conversely, in patients with risk factors and positive biomarkers (BDG), we may initiate anticipatory treatment, though this approach is subject to greater controversy, particularly regarding the cost-effectiveness of biomarkers for diagnosis. Finally, in high-risk patients with fever or shock and an unclear diagnosis, initiating empirical treatment may be a consideration.

The second question pertains to *the most appropriate treatment approach*. As outlined in the objectives, the purpose of this review is to provide readers with the tools to understand different aspects of invasive candidiasis and IAC, as well as the PK/PD characteristics of various antifungals. This will empower each physician to develop an empirical treatment protocol for patients at risk of invasive candidiasis/IAC tailored to the local situation and available resources. However, it is essential to review the international guidelines, which aim to serve as a basis for our local adaptation of treatment. As outlined in Table 6, the main guidelines provide treatment recommendations. Echinocandins are the most frequently mentioned drugs as first-line treatment for invasive candidiasis, and no specific recommendations are made for IAC, with the same treatment being accepted. However, as discussed previously, especially in cases where *C. glabrata* is suspected, this may not be the most suitable option. A recent systematic review has concluded that there are no significant differences in clinical efficacy between treatment with L-AMB, echinocandins or voriconazole in critically ill patients with invasive candidiasis. Therefore, the authors suggest that these three types of drugs should be considered for first-line treatment [1,3,4,51,57,67], and the definitive drug should be chosen based on the isolated species and the susceptibility profile. The use of L-AMB as first-line treatment is supported by various authors, based on the low likelihood of resistance development, especially in patients with a history of prior azole use. Conversely, fluconazole may not be the optimal choice for empirical treatment in patients at risk of invasive candidiasis or IAC due to the high frequency of resistance observed in non-albicans species. Therefore, as is standard practice with antibiotic treatment, an empirical broad-spectrum treatment should be initiated and then de-escalated once microbiological results confirm the species’ susceptibility to fluconazole.

**Figure 1:**
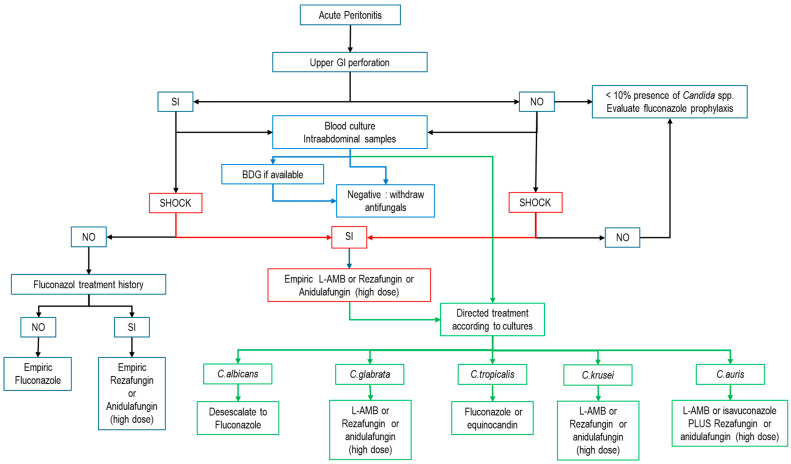
Empirical treatment algorithm for IAC. (BDG: beta-d-glucan; L-AMB: liposomal amphotericin B; modified from refs. [1,51,57,67,85,86].) Blue arrows: possible action according to biomarkers; Red arrows: possible action according to the presence of shock; Green arrows: possible action for targeted therapy.

Finally, Figure 1 proposes an algorithm for the treatment of patients with ICA that can serve as a basis for the development of a local algorithm, depending on the epidemiology, the particular resources of each unit and the species of *Candida* isolated.

## 10. Conclusions

Candidemia and intra-abdominal candidiasis are relatively common complications in critically ill patients associated with high morbidity and mortality. The challenges posed by increasing resistance to antifungal drugs, the need for new and better diagnostic tools, and the development of personalized treatment strategies could improve the grim prognosis of this condition. Therefore, research into new antifungal drugs with different targets and biomarkers that allow more accurate early diagnosis is needed, as well as the development of local and national strategies to control resistance.

## Figures and Tables

**Table 2:** Risk factors for invasive candidiasis in non-neutropenic adult patients (references: [1,2,3,4,5,8]).

Adults		
Patient-Dependent	Treatment-Related	Procedure-Related
Chronic renal failure	Broad-spectrum antibiotics	Abdominal surgery
Severe pancreatitis	Antifungal prophylaxis	Mechanical ventilation
High disease severity	Corticosteroids	Central venous catheter
Diabetes	Parenteral nutrition	
Multifocal colonization	Hemodialysis	
Intra-abdominal bacterial infection	Prolonged ICU stay	

**Table 5:** In vitro susceptibility of available antifungals (++: adequate effect; -/+: possible resistance; --: resistance; L-AMB: liposomal amphotericin B).

Fungal Species	L-AMB	Fluconazole	Voriconazole	Isavuconazole	Echinocandins
*Candida albicans*	++	++	++	++	++
*Candida parapsilosis*	++	++	++	++	+
*Candida tropicalis*	++	++	++	++	++
*Candida glabrata*	++	-/+	-/+	-/+	-/+
*Candida krusei*	++	--	--	--	++
*Candida auris*	+	--	--	++	+

**Table 6:** Summary of recommendations from major international guidelines for empirical treatment in non-neutropenic patients with invasive candidiasis and IAC. (Recommendation A = Strong recommendation for use; B = Moderate recommendation; C = Marginal recommendation; I = High-quality evidence; II = Moderate-quality evidence; III = Expert opinion.)

Invasive Candidiasis	ECIL Recommendation	IDSA Recommendation	ESCMID Recommendation	ESICM/ESCMID Recommendation	ECMM Recommendation
General population	Candins/AI	Candins/strong	Candins/AI	Candins	Candins/AI
	L-AMB/AI	L-AMB/strong	L-AMB/BI	L-AMB as rescue	L-AMB/AI
	Fluconazole/AI	Candins/strong (non-critical patients)	Fluconazole/CI	Fluconazole in patients without DMO	
	Voriconazole/AI	Voriconazole/strong	Voriconazole/BI	Not considered	
IAC	Non-special considerations	Non-special considerations	Non-special considerations		Non-special considerations

ECIL: The European Conference on Infections in Leukemia [85]; IDSA: Infectious Diseases Society of America [57], ESCMID: European Society of Clinical Microbiology and Infectious Diseases [86], ESICM: The European Society of Intensive Care Medicine [51], ECMM: European Confederation of Medical Mycology [67].

## Abbreviations

The following abbreviations are used in this manuscript:

IAC	Intra-abdominal candidiasis
L-AMB	Liposomal amphotericin B
AMB	Amphotericin B
PK/PD	Pharmacokinetic/pharmacodynamic
TDM	Therapeutic drug monitoring
AUC	Area under the curve
MIC	Minimum inhibitory concentration
CYP	Cytochrome
Cmax	Peak plasma concentration
VD	Volume of distribution
